# Gay and bisexual men's awareness and knowledge of treatment as prevention: findings from the Momentum Health Study in Vancouver, Canada

**DOI:** 10.7448/IAS.18.1.20039

**Published:** 2015-08-11

**Authors:** Allison Carter, Nathan Lachowsky, Ashleigh Rich, Jamie I Forrest, Paul Sereda, Zishan Cui, Eric Roth, Angela Kaida, David Moore, Julio SG Montaner, Robert S Hogg

**Affiliations:** 1 Faculty of Health Sciences, Simon Fraser University, Burnaby, BC, Canada; 2 British Columbia Centre for Excellence in HIV/AIDS, Vancouver, BC, Canada; 3 Department of Medicine, Faculty of Medicine, University of British Columbia, Vancouver, BC, Canada; 4 Department of Anthropology, University of Victoria, Victoria, BC, Canada

**Keywords:** treatment as prevention, men who have sex with men, HIV, health literacy, TasP knowledge, TasP awareness

## Abstract

**Introduction:**

Awareness and knowledge of treatment as prevention (TasP) was assessed among HIV-positive and HIV-negative gay, bisexual and other men who have sex with men (GBMSM) in Vancouver, Canada.

**Methods:**

Baseline cross-sectional survey data were analyzed for GBMSM enrolled, via respondent-driven sampling (RDS), in the Momentum Health Study. TasP awareness was defined as ever versus never heard of the term “TasP.” Multivariable logistic regression identified covariates of TasP awareness. Among those aware of TasP, men's level of knowledge of TasP was explored through an examination of self-perceived knowledge levels, risk perceptions and short-answer definitions of TasP which were coded as “complete” if three TasP-related components were identified (i.e. HIV treatment, viral suppression and prevention of transmission). Information source was also assessed. Analyses were stratified by HIV status and RDS adjusted.

**Results:**

Of 719 participants, 23% were HIV-positive, 68% Caucasian and median age was 33 (Interquartile range (IQR) 26,47). Overall, 46% heard of TasP with differences by HIV status [69% HIV-positive vs. 41% HIV-negative GBMSM (*p*<0.0001)]. In adjusted models: HIV-positive GBMSM were more likely to have heard of TasP if they were Canadian born, unemployed, not using party drugs and had higher CD4 counts; HIV-negative GBMSM were more likely to have heard of TasP if they were Caucasian (vs. Aboriginal), students, had higher education, a regular partner and multiple sexual partners. Among those aware of TasP 91% of HIV-positive and 69% of HIV-negative GBMSM (*p*<0.0001) felt they knew “a lot” or “a bit in general” about TasP; 64 and 41% (*p*=0.002) felt HIV treatment made the risk of transmission “a lot lower”; and 21 and 13% (*p*<0.0001) demonstrated “complete” TasP definitions. The leading information source was doctors (44%) for HIV-positive GBMSM and community agencies (38%) for HIV-negative GBMSM, followed by gay media for both populations (34%).

**Conclusions:**

Nearly half of GBMSM in this study reported having heard of TasP, yet only 14% demonstrated complete understanding of the concept. Variations in TasP awareness and knowledge by HIV status, and key socio-demographic, behavioural and clinical factors, highlight a need for health communication strategies relevant to diverse communities of GBMSM in order to advance overall TasP health literacy.

## Introduction

Globally and in Canada, gay, bisexual, and other men who have sex with men (GBMSM) are at high-risk for HIV infection [1]. In British Columbia (BC), Canada, GBMSM comprise 45% of the estimated 9300–13,500 individuals living with HIV and 63% of all new HIV diagnoses in 2012 (150 cases) [2]. The ManCount Survey of GBMSM in Vancouver, the epicentre of BC's epidemic, reported an HIV prevalence of 18% overall, although that figure was approaching one in three for men aged ≥45 years [3].

Treatment as Prevention (TasP) has been actively promoted in Vancouver since 2010, and more recently province-wide, as a critical strategy to reduce HIV morbidity and mortality among individuals living with HIV [4] and at the same time to reduce the transmission of HIV at the population level [5], by lowering viral loads in people with HIV through HIV treatment. This policy, called STOP HIV/AIDS (or “Seek and Treat for Optimal Prevention of HIV/AIDS”), involves the expansion of antiretroviral therapy (ART) to all people living with HIV in BC free-of-charge (for further details: [6]). In 2014, TasP was formally adopted by the United Nations as the global authority's new 90-90-90 strategy (90% diagnosed, 90% on treatment, 90% virally suppressed) to reduce the burden of HIV/AIDS worldwide [7]. As several countries throughout the world incorporate TasP into policy and practice, efforts are needed to understand TasP health literacy among key affected populations.

A 2011–2013 study of TasP among GBMSM in Australia found that, despite generally positive attitudes towards the early initiation of ART, the overwhelming majority (97%) remained sceptical that ART prevented transmission [8]. In qualitative work with people living with HIV in the same setting, despite recognizing the preventive benefits of TasP, participants remained reluctant to take up this approach due to concerns regarding rapidly changing treatment guidelines, the effects of initiating life-long medications, the perception that TasP prioritizes public good over individual agency, and the impact of changing beliefs about infectiousness on people's personal approaches to managing risk and prevention [9]. Similar barriers to TasP acceptability were found in the United Kingdom with inequalities in TasP awareness and literacy levels observed by serostatus; for example, HIV-negative men were less likely to understand key concepts such as the meaning of undetectable viral load and its link to HIV transmission [10]. These findings raise questions about the possible limits of TasP under real world conditions if levels of community awareness and knowledge of TasP are relatively low.

The primary objective of this study was to examine the prevalence of awareness of TasP and analyze associations with key socio-demographic, clinical, and behavioural variables among HIV-positive and HIV-negative GBMSM in Vancouver. Among those aware of TasP, we also examined men's current level of knowledge of TasP, exploring how GBMSM access, understand and perceive this information. To our knowledge, this is the first study in Canada to provide an estimate of TasP awareness and knowledge among GBMSM living with and at-risk for HIV in a setting where a natural experiment for TasP has taken place.

## Methods

### Study population

Baseline cross-sectional data were analyzed for participants enrolled in the Momentum Health Study, a longitudinal bio-behavioural prospective cohort study of HIV-positive and HIV-negative GBMSM (≥16 years) in Vancouver, Canada. Data were collected at participants’ first study visit between February 2012 and February 2014.

### Recruitment and study procedures

Respondent-driven sampling (RDS) was used to recruit GBMSM in the Greater Vancouver area [11]. A computer-assisted, self-administrated (CASI) questionnaire was used to collect socio-demographic and behavioural variables. The CASI was completed at a private computer booth in a study office located in Vancouver's West End traditional gay neighbourhood. Data regarding family doctor and any disclosure to this provider regarding sexual identity and same-sex behaviour were collected through a nurse-administered questionnaire. Data on HIV viral load and CD4 cell count were provided through linkage with administrative data at the British Columbia Centre for Excellence in HIV/AIDS [12]. Participants received honouraria of $50 for completing the study visit (paid in cash and/or prize draw entries for travel or electronics gift cards) and $10 for each person they successfully recruited into the study.

### Outcomes: TasP awareness and knowledge

**Figure 1:**
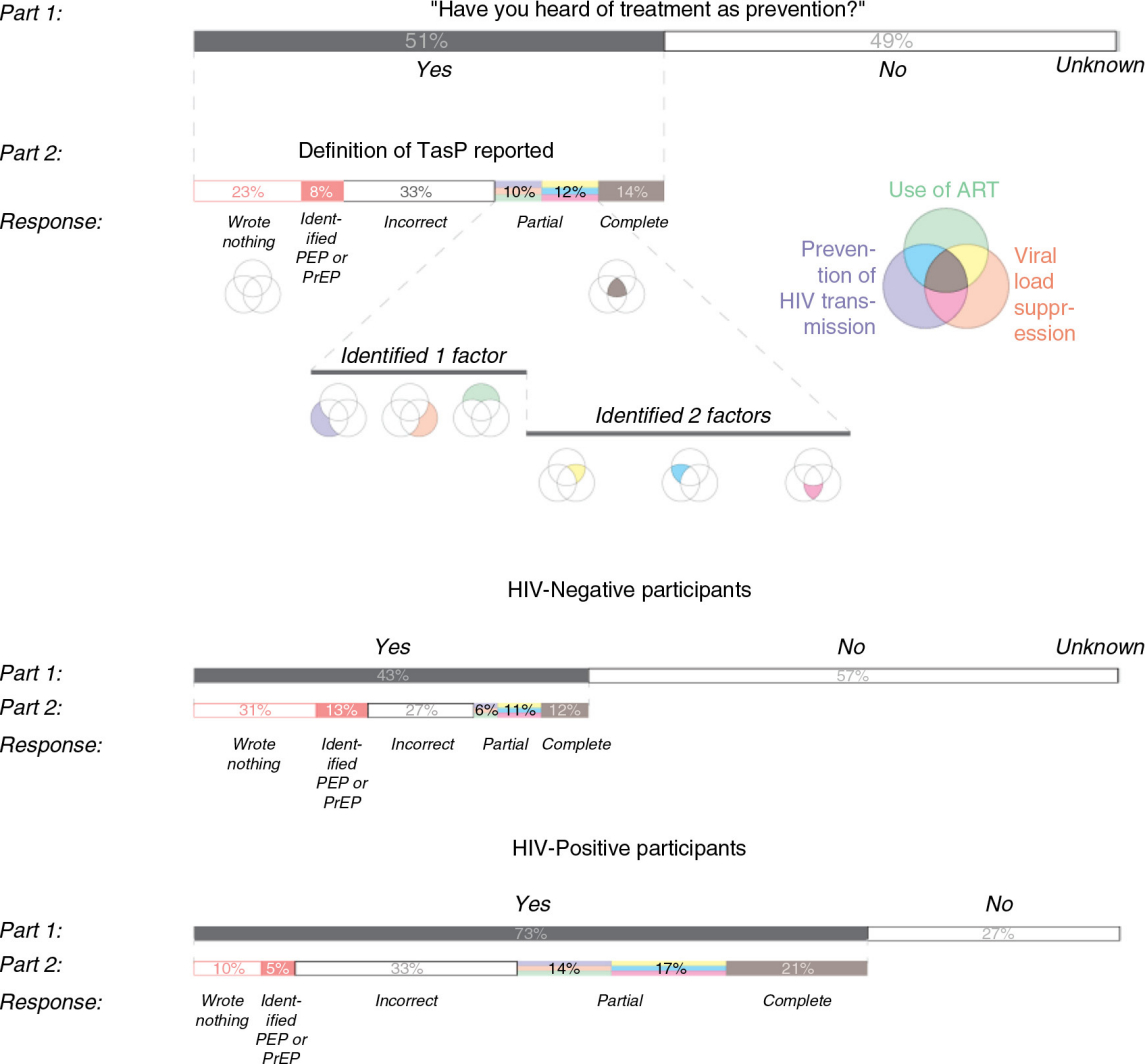
Classification of participants’ self-reported definitions of TasP.

The primary outcome in this study was TasP awareness (ever vs. never heard of the term), assessed through the following question: *Have you ever heard of the term “TasP”?* Among those aware of TasP, participants were then asked how much they thought they knew about TasP, from whom or where they learnt about it, and to provide a definition in their own words (TasP knowledge) (Figure 1). Definitions were prompted using the following question stem: *Please could you give a brief description of what you understand “TasP” to be*. Responses were qualitatively assessed for completeness using a pre-determined three-part definition, developed using TasP literature [13] and refined through a sample of responses to determine appropriate language and scope [14]. The three components of a “complete” definition of TasP included: ART use, viral suppression, and prevention of HIV transmission [among HIV-positive people, as compared with PrEP/PEP (pre-exposure prophylaxis/post-exposure prophylaxis) used in those at-risk]. As shown in Figure 1, these data were then re-coded for quantitative analysis of participant's extent of knowledge, with responses re-coded as complete TasP knowledge (three factors identified), partial TasP knowledge (one or two factors identified), or incorrect TasP knowledge (no factors identified). We also coded those who wrote nothing or described PrEP/PEP.

### Independent variables of interest

Independent variables included socio-demographics (age, race/ethnicity, sexual orientation, highest formal education attained, current student status, country of birth, first language, current neighbourhood, employment status, income, and regular partnership status), behavioural factors [any drug use, party drug use (Cocaine, Crystal, Mushrooms, Nitrous Oxide, LSD, Other Hallucinogens, GHB, Ketamine, Ecstasy, Poppers), injection drug use, number of male anal sex partners in the past six months, any condom-less anal intercourse with a male partner of unknown HIV status, any work as an escort or in the sex industry], and clinical variables (most recent CD4 cell count and viral load).

### Statistical analyses

All analyses were conducted using SAS^®^ version 9.3 (SAS, North Carolina, United States) and adjusted by weights generated using RDSAT version 7.1.46 to better reflect population estimates. Descriptive statistics include crude frequencies and RDS-adjusted proportions. Bivariate and multivariable logistic regression was used to identify covariates of TasP awareness, stratified by HIV status. Model selections were conducted using a backward stepwise elimination technique based on two criteria [Akaike Information Criterion (AIC) and Type III *p*-values] until the final model reached the optimum (minimum) AIC [15]. All statistical tests were two-sided and considered significant at *α*<0.05.

### Ethical statement

All participants provided voluntary informed consent at study enrolment. The Research Ethics Boards of Simon Fraser University, University of British Columbia/Providence Health, and the University of Victoria provided ethical approval for all study procedures.

## Results

A total of 719 participants were included in this study, of whom 119 (17%) were recruited as seeds. After RDS-adjustment, 23% of this total were HIV-positive, 68% were Caucasian, 81% identified as gay and median age was 33 [IQR 26,47]. Overall, 86% of the sample reported high school education or greater, 52% were currently employed, 53% had annual incomes <$18,500, 34% reported a current regular partner, 75% were born in Canada and 52% lived in Vancouver's downtown/West End area, which is the historic neighbourhood with a substantial gay men's population. Other demographics are shown in Table 1. Among HIV-positive participants, 89% were receiving ART, of whom 67% were ≥95% adherent to treatment in the past six months (based on pharmacy refill data), 83% had a CD4 ≥350 cells/mm^3^ and 72% had an undetectable VL (<50 copies/mL). From this total, two men refused to answer TasP questions and were excluded from subsequent analyses.

**Table 1:** Sample demographics

	*n*	RDS %	RDS (95% CI)
HIV positive			
No	520	76.6	(68.7, 83.9)
Yes	199	23.4	(16.1, 31.3)
Age			
16–24	139	21.4	(15.0, 28.7)
25–39	305	41.9	(34.7, 48.7)
40+	275	36.7	(28.2, 45.4)
Ethnicity			
Caucasian	539	68.0	(61.0, 74.2)
Asian	72	9.8	(6.3, 14.7)
Aboriginal ancestry	50	10.3	(5.5, 15.9)
Other	58	11.9	(7.3, 17.0)
Sexual orientation			
Gay	612	80.7	(76.2, 85.3)
Bisexual	66	15.3	(10.6, 19.5)
Other	41	4.0	(2.4, 6.2)
Education			
Some high school or less	61	14.5	(10.1, 20.8)
Completed high school (only)	107	20.2	(14.5, 25.0)
Any post-secondary education	537	65.3	(58.0, 72.3)
Current student			
No	568	81.0	(75.9, 86.0)
Yes	150	19.0	(14.0, 24.1)
Born in Canada			
No	162	25.3	(19.5, 32.1)
Yes	557	74.7	(67.9, 80.5)
First language			
English	597	79.1	(72.9, 84.7)
Other	122	20.9	(15.3, 27.1)
Neighbourhood			
Downtown/West End	356	51.9	(44.1, 59.2)
Elsewhere in Vancouver	223	30.4	(24.1, 36.3)
Outside Vancouver	140	17.7	(13.1, 23.7)
Currently employed			
No	264	48.0	(41.3, 55.2)
Yes	455	52.0	(44.8, 58.7)
Income			
<$18,500	328	52.5	(46.2, 59.2)
$18,500–44,999	247	32.8	(26.9, 38.3)
$50,000–74,999	101	9.4	(6.2, 12.8)
$75,000+	43	5.3	(2.8, 8.1)
Relationship with regular partner			
No	446	65.6	(58.4, 71.5)
Yes	232	34.4	(28.5, 41.6)
Any reported drug use in the past 6 months			
No	258	34.7	(28.9, 41.2)
Yes	461	65.3	(58.8, 71.1)
Any reported party drug use in the past 6 months			
No	288	40.7	(34.6, 46.7)
Yes	431	59.3	(53.3, 65.4)
Any reported injection drug use in the past 6 months			
No	662	90.5	(86.3, 94.5)
Yes	57	9.5	(5.5, 13.7)
Number of male anal sex partners in the past 6 months			
0–1	229	35.0	(29.1, 41.6)
2–5	208	25.7	(21.2, 31.3)
6+	195	25.6	(19.0, 30.7)
No anal sex in the past 6 months	87	13.8	(9.9, 18.4)
Unprotected anal sex with opposite or unknown status partner			
No	441	64.1	(58.0, 70.7)
Yes	262	35.9	(29.3, 42.0)
Worked as an escort or in the sex industry			
No	588	79.4	(73.6, 84.6)
Yes, in the past 6 months	43	8.5	(4.5, 13.3)
Yes, but not in the past 6 months	88	12.1	(8.3, 16.3)
Current CD4 cell count			
<200	13	6.6	(2.3, 11.8)
200–349	23	11.6	(4.5, 23.0)
350+	159	81.7	(69.2, 90.8)
Current viral load <50			
No	60	28.1	(19.6, 45.2)
Yes	139	71.9	(54.8, 80.4)
Currently has a family doctor			
No	232	34.2	(27.4, 41.3)
Yes	486	65.8	(58.7, 72.6)
Out to family doctor			
No	80	18.8	(12.5, 27.9)
Yes	400	81.2	(72.1, 87.5)
Told family doctor about male partners			
No family doctor	232	34.9	(28.5, 42.3)
Did not tell	80	14.6	(10.5, 19.5)
Told doctor	400	50.5	(43.1, 57.5)

RDS=respondent-driven sampling; 95% CI=95% confidence interval.

### TasP awareness

Overall, 46% of GBMSM had heard of TasP. HIV-positive men were more likely to have heard of TasP (69%) compared with HIV-negative men (41%, *p*<0.0001). Tables 2 and 3 show the RDS-adjusted demographic and risk factors, prevalence of TasP awareness and univariate associations for HIV-positive GBMSM (*n*=199) and HIV-negative GBMSM (*n*=520), respectively. The adjusted multivariable logistic regression models stratified by HIV status are shown in Table 4. In the adjusted models, among *HIV-positive* GBMSM, TasP awareness was significantly *higher* among men born in Canada (vs. not) [AOR (95% CI)=4.05 (1.52–10.80)] and men with a current CD4 cell count of ≥350 (vs. <200) [6.30 (1.30–30.64)]; and significantly *lower* among men who identified as bisexual (vs. gay) [0.15 (0.05–0.47)], currently employed (vs. not) [0.28 (0.13–0.62)] and had used any party drugs in the past six months (vs. none) [0.35 (0.13–0.95)]. Among *HIV-negative* GBMSM, TasP awareness was significantly *higher* among men who completed high school [3.33 (1.40–7.95)] or any post-secondary education [3.49 (1.60–7.61)] (vs. some or no high school), were a current student (vs. not) [1.67 (1.09–2.59)], had a regular partner (vs. not) [1.91 (1.27–2.87)] and had ≥6 [1.94 (1.07–3.52)] or 2–5 [1.77 (1.06–2.95)] male anal sex partners in the past six months (vs. 0–1 partners); and significantly *lower* among men who identified as bisexual (vs. gay) [0.45 (0.24–0.85)] and Aboriginal (vs. Caucasian) [0.38 (0.15–0.97)].

**Table 2:** Demographic and risk factors, prevalence of TasP awareness and univariate associations for *HIV-positive* GBMSM (*n*=199)

	Total (HIV positive)			Aware of TasP			Univariate associations	
			
	*n*	RDS %	RDS (95% CI)	*n*	RDS %	RDS (95% CI)	*p*	OR (95% CI)
Age								
16–24	0	0						
25–39	48	24.4	(15.9, 32.9)	34	77.4	(62.9, 92.0)	0.1379	Reference
40+	151	75.6	(67.1, 84.1)	110	64.8	(52.1, 77.5)		0.54 (0.24–1.22)
Ethnicity								
Caucasian	150	67.5	(56.9, 78.1)	115	71.5	(59.8, 83.3)	0.5860	Reference
Asian	13	6.7	(2.5, 10.9)	8	62.3	(27.4, 97.1)		0.66 (0.18–2.34)
Aboriginal ancestry	23	17.1	(8.0, 26.2)	12	61.0	(30.2, 91.8)		0.62 (0.27–1.46)
Other	13	8.7	(1.5, 16.0)	9	59.5	(7.8, 100.0)		0.58 (0.19–1.78)
Sexual orientation								
Gay	171	83.0	(74.6, 91.4)	128	72.1	(61.1, 83.1)	0.0139	Reference
Bisexual	21	12.5	(4.8, 20.2)	11	39.1	(3.5, 74.7)		0.25 (0.10–0.64)
Other	7	4.5	(0.5, 8.6)	5	76.0	(32.7, 100.0)		1.23 (0.22–6.76)
Education								
Some high school or less	24	13.4	(6.5, 20.4)	14	54.9	(29.0, 80.7)	0.4093	Reference
Completed high school (only)	39	20.8	(11.5, 30.0)	27	64.6	(39.2, 90.0)		1.50 (0.47–4.80)
Any post-secondary education	132	65.8	(55.5, 76.1)	99	70.3	(57.3, 83.3)		1.95 (0.71–5.37)
Current student								
No	180	90.5	(85.4, 95.6)	129	67.6	(56.4, 78.7)	0.7547	Reference
Yes	19	9.5	(4.4, 14.6)	15	71.4	(44.5, 98.3)		1.20 (0.38–3.75)
Born in Canada								
No	35	17.3	(9.2, 25.4)	22	41.8	(17.0, 66.6)	0.0012	Reference
Yes	164	82.7	(74.6, 90.8)	122	73.7	(63.4, 84.0)		3.91 (1.71–8.90)
First language								
English	165	83.0	(74.9, 91.1)	121	72.5	(62.2, 82.8)	0.0087	Reference
Other	34	17.0	(8.9, 25.1)	23	46.8	(19.9, 73.6)		0.33 (0.15–0.76)
Neighbourhood								
Downtown/West End	136	68.5	(59.0, 78.0)	95	65.7	(52.7, 78.7)	0.4996	Reference
Elsewhere in Vancouver	37	18.8	(10.5, 27.2)	29	68.5	(42.6, 94.4)		1.14 (0.49–2.64)
Outside Vancouver	26	12.7	(6.8, 18.6)	20	78.9	(60.4, 97.3)		1.95 (0.64–5.92)
Currently employed								
No	116	60.9	(50.6, 71.2)	86	79.1	(69.9, 88.2)	0.0003	Reference
Yes	83	39.1	(28.8, 49.4)	58	51.5	(34.1, 69.0)		0.28 (0.14–0.56)
Income								
<$18,500	114	61.1	(50.7, 71.4)	78	67.5	(55.3, 79.7)	0.9353	Reference
$18,500–44,999	55	25.5	(16.0, 34.9)	42	66.6	(42.9, 90.2)		0.96 (0.45–2.05)
$50,000–74,999	25	12.7	(6.1, 19.4)	20	71.3	(36.1, 100.0)		1.20 (0.43–3.36)
$75,000+	5	0.7	(0.0, 1.6)	4	92.7	(68.2, 100.0)		n/a
Relationship with regular partner								
No	125	71.2	(61.4, 81.1)	86	66.8	(53.6, 80.0)	0.7135	Reference
Yes	55	28.8	(18.9, 38.6)	42	70.0	(49.4, 90.5)		1.16 (0.53–2.53)
Any reported drug use in the past 6 months								
No	44	16.5	(10.2, 22.7)	36	82.6	(69.4, 95.8)	0.0786	Reference
Yes	155	83.5	(77.3, 89.8)	108	65.0	(53.0, 77.0)		0.39 (0.14–1.11)
Any reported party drug use in the past 6 months								
No	56	24.8	(16.4, 33.1)	44	82.1	(70.2, 94.0)	0.0280	Reference
Yes	143	75.2	(66.9, 83.6)	100	63.2	(50.4, 76.0)		0.37 (0.16–0.90)
Any reported injection drug use in the past 6 months								
No	168	87.8	(81.4, 94.1)	128	68.5	(57.0, 80.0)	0.6849	Reference
Yes	31	12.2	(5.9, 18.6)	16	64.1	(40.2, 87.9)		0.82 (0.31–2.15)
Number of male anal sex partners in the past 6 months								
0–1	55	30.6	(20.9, 40.3)	41	72.7	(54.9, 90.5)	0.5790	Reference
2–5	52	22.3	(14.5, 30.1)	40	71.9	(55.7, 88.0)		0.96 (0.36–2.45)
6+	69	32.4	(22.0, 42.8)	49	64.6	(43.6, 85.7)		0.69 (0.30–1.59)
No anal sex in the past 6 months	23	14.7	(6.7, 22.7)	14	58.9	(26.3, 91.5)		0.54 (0.20–1.47)
Unprotected anal sex with opposite or unknown status partner								
No	107	54.9	(44.2, 65.6)	77	70.4	(57.9, 83.0)	0.8183	Reference
Yes	88	45.1	(34.4, 55.8)	66	68.7	(52.2, 85.3)		0.92 (0.47–1.83)
Worked as an escort or in the sex industry								
No	136	67.0	(56.7, 77.3)	101	67.8	(55.1, 80.5)	0.4891	Reference
Yes, in the past 6 months	15	6.4	(0.8, 12.0)	12	83.8	(58.5, 100.0)		2.45 (0.46–12.99)
Yes, but not in the past 6 months	48	26.6	(16.8, 36.3)	31	64.1	(42.4, 85.9)		0.85 (0.40–1.80)
Current CD4 cell count								
<200	13	5.4	(1.5, 9.3)	5	31.9	(0.0, 74.3)	0.0729	Reference
200–349	23	12.5	(4.7, 20.2)	17	66.0	(29.2, 100.0)		4.16 (0.80–21.66)
350+	159	82.1	(73.7, 90.5)	119	71.1	(59.8, 82.4)		5.27 (1.26–22.06)
Current viral load <50								
No	60	29.0	(19.0, 39.0)	37	65.6	(46.2, 85.0)	0.6724	Reference
Yes	139	71.0	(61.0, 81.0)	107	69.0	(56.5, 81.4)		1.17 (0.58–2.37)
Currently has a family doctor								
No	6	4.7	(0.0, 10.0)	2	65.5	(0.7, 100.0)	0.8802	Reference
Yes	193	95.3	(90.0, 100.0)	142	68.1	(57.4, 78.7)		1.12 (0.25–5.08)
Out to family doctor								
No	9	5.2	(1.3, 9.2)	4	46.7	(0.0, 94.6)	0.1903	Reference
Yes	182	94.8	(90.8, 98.7)	136	69.1	(57.9, 80.2)		2.54 (0.63–10.29)
Told family doctor about male partners								
No family doctor	6	4.7	(0.0, 10.1)	2	65.5	(0.7, 100.0)	0.4203	Reference
Did not tell	9	5	(1.2, 8.8)	4	46.7	(0.0, 94.6)		0.46 (0.06–3.41)
Told doctor	182	90.3	(83.9, 96.7)	136	69.1	(57.9, 80.2)		1.18 (0.26–5.34)

GBMSM=gay, bisexual, and other men who have sex with men; RDS= respondent-driven sampling; 95% CI=95% confidence interval.

**Table 3:** Demographic and risk factors, prevalence of TasP awareness and univariate associations for *HIV-negative* GBMSM (*n*=520)

	Total (HIV positive)			Aware of TasP			Univariate associations	
			
	*n*	RDS %	RDS (95% CI)	*n*	RDS %	RDS (95% CI)	*p*	OR (95% CI)
Age								
16–24	139	27.5	(22.1, 32.9)	52	31.6	(21.1, 42.2)	0.1905	Reference
25–39	257	48.5	(42.4, 54.6)	113	39.9	(31.3, 48.5)		1.44 (0.95–2.18)
40+	124	24.0	(18.8, 29.2)	57	40.2	(28.3, 52.1)		1.45 (0.89–2.37)
Ethnicity								
Caucasian	389	70.1	(64.1, 76.0)	173	37.9	(31.3, 44.4)	0.0343	Reference
Asian	59	11.0	(7.5, 14.4)	19	41.3	(24.1, 58.6)		1.16 (0.67–2.00)
Aboriginal ancestry	27	6.8	(3.1, 10.4)	8	16.4	(2.0, 30.9)		0.32 (0.13–0.77)
Other	45	12.2	(7.5, 16.9)	22	45.3	(23.5, 67.1)		1.36 (0.81–2.29)
Sexual orientation								
Gay	441	82.0	(77.1, 86.9)	186	39.4	(33.0, 45.9)	0.0042	Reference
Bisexual	45	13.3	(8.6, 18.0)	16	21.2	(8.1, 34.3)		0.41 (0.23–0.75)
Other	34	4.7	(2.9, 6.5)	20	52.8	(32.3, 73.3)		1.72 (0.77–3.83)
Education								
Some high school or less	37	11.7	(7.0, 16.4)	12	14.2	(3.1, 25.3)	0.0004	Reference
Completed high school (only)	68	17.2	(12.1, 22.3)	23	35.4	(19.2, 51.7)		3.31 (1.45–7.58)
Any post-secondary education	405	71.1	(65.0, 77.2)	183	42.0	(35.4, 48.5)		4.37 (2.09–9.15)
Current student								
No	388	74.5	(69.3, 79.7)	160	34.7	(28.2, 41.3)	0.0153	Reference
Yes	131	25.5	(20.3, 30.7)	62	46.3	(34.5, 58.1)		1.62 (1.10–2.39)
Born in Canada								
No	127	28.4	(22.7, 34.2)	50	40.7	(28.5, 52.9)	0.3529	Reference
Yes	393	71.6	(65.8, 77.3)	172	36.4	(30.0, 42.9)		0.84 (0.57–1.22)
First language								
English	432	78.9	(73.5, 84.3)	193	36.7	(30.6, 42.9)	0.3962	Reference
Other	88	21.1	(15.7, 26.5)	29	41.0	(26.0, 56.1)		1.20 (0.80–1.82)
Neighbourhood								
Downtown/West End	220	45.0	(38.9, 51.2)	97	40.7	(31.2, 50.2)	0.2786	Reference
Elsewhere in Vancouver	186	31.4	(26.1, 36.8)	86	37.2	(28.1, 46.4)		0.86 (0.58–1.29)
Outside Vancouver	114	23.6	(18.5, 28.6)	39	32.4	(20.6, 44.2)		0.70 (0.45–1.09)
Currently employed								
No	148	36.8	(30.6, 43.0)	58	29.4	(19.8, 39.0)	0.0024	Reference
Yes	372	63.2	(57.0, 69.4)	164	42.5	(35.5, 49.5)		1.77 (1.22–2.56)
Income								
<$18,500	214	47.0	(40.9, 53.2)	91	37.1	(28, 46.2)	0.9759	Reference
$18,500–44,999	192	35.2	(29.5, 40.9)	81	37.8	(28.4, 47.2)		1.03 (0.70–1.51)
$50,000–74,999	76	9.8	(6.7, 12.8)	34	40.3	(25, 55.6)		1.15 (0.63–2.09)
$75,000+	38	8.0	(4.8, 11.2)	16	37.1	(17.1, 57)		0.10 (0.52–1.93)
Relationship with regular partner								
No	321	65.9	(60.2, 71.7)	129	34.4	(27.2, 41.7)	0.0109	Reference
Yes	177	34.1	(28.3, 39.8)	85	45.9	(35.7, 56.1)		1.61 (1.12–2.33)
Any reported drug use in the past 6 months								
No	214	43.5	(37.5, 49.6)	94	43.6	(34.5, 52.7)	0.0114	Reference
Yes	306	56.5	(50.4, 62.5)	128	33.1	(25.8, 40.3)		0.64 (0.45–0.90)
Any reported party drug use in the past 6 months								
No	232	47.4	(41.3, 53.5)	99	41.5	(32.7, 50.2)	0.0826	Reference
Yes	288	52.6	(46.5, 58.7)	123	34.3	(26.7, 41.9)		0.74 (0.52–1.04)
Any reported injection drug use in the past 6 months								
No	494	94.3	(90.8, 97.9)	212	38.7	(32.8, 44.7)	0.0388	Reference
Yes	26	5.7	(2.1, 9.2)	10	20.0	(1.7, 38.2)		0.40 (0.16–0.95)
Number of male anal sex partners in the past 6 months								
0–1	174	35.2	(29.3, 41.1)	71	34.5	(24.8, 44.1)	0.0365	Reference
2–5	156	29.2	(24.0, 34.4)	60	31.9	(22.5, 41.3)		0.90 (0.57–1.39)
6+	126	22.9	(17.4, 28.3)	58	44.2	(30.3, 58.0)		1.50 (0.95–2.38)
No anal sex in the past 6 months	64	12.7	(8.8, 16.6)	33	48.1	(31.2, 65.0)		1.76 (1.01–3.07)
Unprotected anal sex with opposite or unknown status partner								
No	334	68.9	(63.4, 74.5)	146	35.6	(28.6, 42.6)	0.0927	Reference
Yes	174	31.1	(25.5, 36.6)	74	43.2	(32.7, 53.7)		1.38 (0.95–2.00)
Worked as an escort or in the sex industry								
No	452	85.6	(81.1, 90.2)	195	40.0	(33.6, 46.3)	0.0070	Reference
Yes, in the past 6 months	28	7.9	(3.8, 12.0)	10	14.8	(2.2, 27.4)		0.26 (0.11–0.60)
Yes, but not in the past 6 months	40	6.5	(4.1, 8.9)	17	35.7	(17.9, 53.5)		0.83 (0.41–1.69)
Currently has a family doctor								
No	226	44.9	(38.8, 51)	84	37.5	(28.1, 46.8)	0.9496	Reference
Yes	293	55.1	(49, 61.2)	137	37.7	(30.4, 45)		1.01 (0.72–1.43)
Out to family doctor								
No	71	32.3	(24.4, 40.2)	30	33.7	(19.7, 47.7)	0.2996	Reference
Yes	218	67.7	(59.8, 75.6)	105	39.9	(31.3, 48.6)		1.31 (0.79–2.18)
Told family doctor about male partners								
No family doctor	226	45.2	(39.1, 51.4)	84	37.5	(28.1, 46.8)	0.5793	Reference
Did not tell	71	17.7	(12.7, 22.6)	30	33.7	(19.7, 47.7)		0.85 (0.52–1.39)
Told doctor	218	37.1	(31.4, 42.8)	105	39.9	(31.3, 48.6)		1.11 (0.76–1.62)

GBMSM=gay, bisexual, and other men who have sex with men; RDS= respondent-driven sampling; 95% CI=95% confidence interval.

**Table 4:** Multivariable models of TasP awareness stratified by HIV status

	HIV-negative GBMSM AOR (95% CI)	HIV-positive GBMSM AOR (95% CI)
Ethnicity		
Caucasian	Reference	
Asian	0.91 (0.51–1.63)	
Aboriginal ancestry	0.38 (0.15–0.97)	
Other	1.42 (0.81–2.49)	
Sexual orientation		
Gay	Reference	Reference
Bisexual	0.45 (0.24–0.85)	0.15 (0.05–0.47)
Other	1.75 (0.75–4.11)	0.71 (0.10–5.21)
Education		
Some high school or less	Reference	
Completed high school (only)	3.33 (1.40–7.95)	
Any post-secondary education	3.49 (1.60–7.61)	
Current student		
No	Reference	
Yes	1.67 (1.09–2.58)	
Born in Canada		
No		Reference
Yes		4.05 (1.52–10.80)
Currently employed		
No		Reference
Yes		0.28 (0.13–0.62)
Relationship with regular partner		
No	Reference	
Yes	1.91 (1.27–2.87)	
Any reported party drug use in the past 6 months		
No		Reference
Yes		0.35 (0.13–0.95)
Number of male anal sex partners in the past 6 months		
0–1	Reference	
2–5	0.75 (0.46–1.21)	
6+	1.77 (1.06–2.95)	
No anal sex in the past 6 months	1.94 (1.07–3.52)	
Current CD4 cell count		
<200		Reference
200–349		4.12 (0.69–24.64)
350+		6.30 (1.30–30.64)

GBMSM=gay, bisexual, and other men who have sex with men; RDS=respondent-driven sampling; 95% CI=95% confidence interval.

### TasP knowledge

Among HIV-positive (*n*=144) and HIV-negative (*n*=222) GBMSM aware of TasP, Table 5 presents their self-perceived knowledge level, risk perceptions and information source. After RDS-adjustment, 91% of HIV-positive men who had heard of TasP felt they knew “a lot” or “a bit in general” about TasP compared with 69% of HIV-negative men who had heard of TasP (*p*<0.0001). In addition, 64 and 41%, respectively, felt HIV treatment made the risk of transmitting or acquiring HIV “a lot lower” (*p*=0.0020). The leading information sources for HIV-positive GBMSM were doctors (44%) (vs. 10% for HIV-negative men, *p*<0.0001) and community agencies (38%) (vs. 25% for HIV-negative men, *p*=0.0338). Gay media was also an important information source for men regardless of HIV status (34% for both HIV-positive and HIV-negative men, *p*=0.9517). Other sources included friends (20% HIV-positive vs. 32% HIV-negative men, *p*=0.0392) and sex partners (10% vs. 17%, *p*=0.1211).

**Table 5:** Self-perceived knowledge, source of awareness and impact on HIV transmission of Treatment as Prevention (TasP)

	Total sample			HIV-negative GBMSM			HIV-positive GBMSM			
				
	*n*	RDS %	RDS (95% CI)	*n*	RDS %	RDS (95% CI)	*n*	RDS %	RDS (95% CI)	*p*
How much do you think you know about what TasP means? (*n*=366)										<0.0001
Not much, or nothing at all	77	20.0	(10.0, 28.5)	66	30.6	(22.4, 38.8)	11	9.5	(1.0, 17.9)	
A bit in general	201	57.1	(47.0, 68.6)	126	57.1	(48.1, 66.0)	75	52.4	(40.0, 64.8)	
A lot	88	22.9	(14.3, 33.8)	30	12.3	(7.2, 17.4)	58	38.1	(26.2, 50.1)	
Who or where did you learn about TasP from? (*n*=289) [all that apply]										
Friends	84	27.6	(13.7, 38.6)	56	31.5	(20.8, 42.2)	28	19.8	(9.2, 30.4)	0.0392
Sex partners	40	15.8	(6.0, 25.9)	21	17.1	(7.4, 26.9)	19	10.2	(1.6, 18.9)	0.1211
Community agency	106	31.7	(29.1, 55.4)	47	25.3	(15.8, 34.8)	59	37.7	(25.6, 49.8)	0.0338
Doctor	76	27.8	(16.5, 41.4)	17	9.6	(4.2, 14.9)	59	44.0	(31.4, 56.7)	<0.0001
Gay media	102	31.1	(25.1, 53.2)	57	33.9	(23.1, 44.7)	45	34.2	(21.8, 46.7)	0.9517
How do you think that TasP changes your current risk of getting or transmitting HIV? (*n*=289)										0.0020
A lot lower	143	57.5	(42.6, 69.2)	54	40.5	(28.7, 52.4)	89	63.6	(51.4, 75.8)	
A little lower	86	28.6	(19.4, 45.7)	65	41.0	(29.7, 52.4)	21	19.1	(8.2, 30.1)	
No difference	50	10.9	(4.9, 15.4)	31	16.2	(9.5, 22.9)	19	13.7	(6.5, 20.8)	
A little higher	5	1.4	(0.0, 2.1)	3	0.9	(0, 1.9)	2	2.3	(0, 6.5)	
A lot higher	5	1.6	(0.0, 4.0)	3	1.3	(0, 3.1)	2	1.3	(0, 3.2)	

GBMSM=gay, bisexual, and other men who have sex with men; RDS=respondent-driven sampling; 95% CI=95% confidence interval.

Qualitative analysis of participants’ short-answer definitions of TasP revealed that only 14% of participants who had heard of TasP demonstrated complete TasP knowledge with all three factors identified (ART use, viral suppression and prevention of HIV transmission), while 12% identified two out of three TasP factors, and 43% identified one or none. The remaining men provided no definition (23%) or described PrEP/PEP (7%). By HIV status, 21% of HIV-positive men and 13% of HIV-negative men (*p*<0.0001) identified all three TasP factors. The factor identified most was “ART use” (48% HIV-positive vs. 29% HIV-negative men, *p*<0.0001). The factor omitted most was “viral suppression” (30% HIV-positive vs. 14% HIV-negative men, *p*<0.0001). Figure 1 illustrates the division of TasP definitions.

An illustrative example of a complete definition was reported by a participant who said: *By getting treatment, viral load goes to “non-detectable” (ideally) therefore lessening chances of transmission* (HIV-positive, Caucasian, 52 years). However, the vast majority of men were unable to clearly express a complete understanding of TasP. For example, one participant explained: *The more regular testing you get, the more you are exposed to STI/HIV information/education and the more likely you are to practice safer sex and prevent infections* (HIV-negative, Latin American, 29 years). This incorrect definition does, however, highlight testing, which is one element to the implementation of BC's TasP policy overall. In other cases, men were unable to articulate essential differences between PrEP, PEP and TasP, for example: *Taking the new drug for neg people to use if they have a poz partner or are seeing many poz guys or high risk behaviours* (HIV-negative, Caucasian, 58 years). A sample of participants’ definitions is shown in Table 6.

**Table 6:** A sample of participants’ definitions of TasP

Complete (3 factors identified)	“By being on treatment and getting to undetectable level you chance of spreading the virus drops by 96%.” “I think treatment as prevention is when someone who is HIV+ receives HAART in order to reduce their viral load down to undetectable to prevent others from getting infected with HIV.” “By take ART medication and becoming undetectable is the best way to ensure that I will not pass the bug forward.” “The idea that, in public health terms, if enough people with HIV are on anti-retroviral treatments the scale of new infections will decrease because enough HIV-positive people will have undetectable, and thus incommunicable, viral loads.”	
Two factors identified	“By having more people living with HIV on anti-retroviral therapy, there is significantly less risk of HIV transmission.” “Maximizing treatment of the known HIV+ population will reduce the risk of transmission thereby reducing the number of new cases.” “Basically what I’ve said to my partners: Find out if you’re poz so that you can start treatment ASAP and you’ll be less like to transmit HIV to another person.”	
One factor identified	“Going on the cocktail.” “Taking my medication regularly.” “Treating a person’s HIV with medication and using education to change sexual behaviours.” “De-stigmatize HIV/illness/addiction to enrol people in testing/treatment programmes.”	
Incorrect (No factors identified)	“I don’t really understand it all that well.” “Do everything you can to not pass it on.” “Condoms and safe sex.” “Be very careful in bed.” “Being tested on a regular basis and safe sex.” “Getting tested regularly which is every 3 months for at-risk guys and every 6 months for low-risk guys.” “As I understood it, it had to do with always checking up and getting tested as a form of prevention.”	
PrEP/PEP only	“I heard it could be a pill you take as a treatment to prevent getting HIV.” “Taking the drugs to help you not to contract if exposed.” “The use of P.R.E.P drugs to prevent successfully contracting HIV during risky sex. Moreover, the use of safe sex practices and a general awareness of risk when engaging in various kinds of sex.” “When someone is exposed to the virus by unprotected sex and its confirmed or to create piece of mind ARV’s are used to prevent infection.”	

## Discussion

This cross-sectional survey of GBMSM in Vancouver, Canada, indicates that while TasP awareness was high among HIV-positive men (69%), it was relatively low among HIV-negative men (41%) and varied by key socio-demographic, clinical and behavioural factors among both populations. Further, men's articulation of their knowledge of TasP was poor, albeit better among HIV-positive men. To our knowledge, this is first study to provide an estimate of TasP awareness and knowledge among GBMSM living with and at-risk for HIV in a setting where a natural experiment for TasP has taken place, and the results have important implications for HIV care, prevention and education in BC and globally as jurisdictions scale-up the implementation of TasP into practice.

We suspect that some of the differences in TasP awareness and knowledge observed by HIV status are because TasP messaging and practices are largely targeted and taken by people with HIV as they are the recipient of ART in this strategy. The personal health benefits of ART for HIV-positive people, in terms of reduced morbidity and mortality [4], may explain some of the difference in incentive for HIV-positive GBMSM to learn about TasP. Indeed, in previous research [8–10], HIV-negative GBMSM have been shown to demonstrate lower TasP literacy with a lack of understanding of undetectable viral loads and scepticism that highly active antiretroviral therapy (HAART) prevents transmission. The benefits of TasP for HIV-negative people, in terms of prevention of transmission [16], require that information on TasP be made available and accessible to diverse communities of GBMSM irrespective of HIV status. As bioethicists have highlighted, “a treatment-as-prevention strategy that places all the emphasis upon the positive person's adherence … carries a disproportionate burden of responsibility” [17, pp. 63]. TasP is an important strategy in the arsenal of HIV prevention tools for all men, along with access to a combination of other evidence-based biomedical (e.g. PrEP/PEP), behavioural (e.g. consistent and correct use of condoms and lubricant), and structural (e.g. reducing stigma) HIV prevention interventions [18,19]. Meaningful engagement of HIV uninfected men in TasP initiatives are critical so that they can incorporate this information into their sexual decision-making and support their own health and the health of their partners and communities. Within this context, it is important to understand how HIV-negative versus HIV-positive GBMSM differentially access, perceive, and use TasP information, with special consideration given to men's own personal risk reduction strategies as well as the wider barriers to TasP such as the criminalization of HIV transmission, widespread stigma, and other social constraints [8–10].

A patient's health literacy can play an important role in overall health and clinical outcomes across many health issues [20]. In our study, HIV-positive GBMSM with higher CD4 cell counts were more likely to be aware of TasP; however, no association was found between TasP awareness and ART adherence or viral suppression. In addition, factors associated with increased HIV transmission were investigated in this study, with different patterns found by HIV status. For HIV-negative men, reporting two or more recent male anal sex partners was positively associated with TasP awareness. However, among HIV-positive men, any party drug use was negatively associated with TasP awareness, suggesting a greater lack of awareness of TasP among those with a potential greater risk of HIV transmission. There is concern that the public health benefits of TasP could be overwhelmed by increased risk behaviours, commonly referred to as risk compensation [21]. However, recent intervention research with GBMSM has demonstrated that exposure to multiple messages regarding HIV prevention strategies (PrEP/PEP, rectal microbicides) did not affect men's intentions to use condoms nor their attitudes regarding unprotected sex [22]. This is consistent with other studies of ART [23] and PrEP/PEP [24,25], which have reported no evidence of risk compensation that would offset the benefits of using HIV treatment as an effective prevention strategy. Future research will be conducted using longitudinal Momentum Health Study data to explore the relationship between TasP awareness and knowledge, treatment optimism, and risk compensation in this population.

Consistent with previous research [26,27], study findings also indicate important cultural and structural barriers to access to information regarding TasP. For example, HIV-negative Aboriginal men were less likely than their Caucasian counterparts to be aware of TasP as were HIV-positive men not born in Canada and HIV-negative men without high school education, highlighting a need for TasP messaging that is culturally relevant, responsive to literacy levels, and aware of other barriers in health care. Further, for HIV-positive men, TasP awareness was associated with unemployment. While this may seem counterintuitive, we suspect that this may be linked to men who have been living with HIV for longer (and thus likely more aware of issues such as TasP) and who have removed themselves from the job market to deal with their illness. Finally, regardless of HIV status, gay men were more likely to have heard of TasP than bisexual men. Previous research regarding biomedical approaches to HIV prevention has demonstrated the need for increased levels of community education to raise awareness and capacity within communities of GBMSM [28]. The differential access to and uptake of health promotion messaging among bisexual and other non-gay identified GBMSM in this study must be considered in future education campaigns and interventions.

Among those aware of TasP, despite a majority reporting that they felt they knew “a lot” or “a bit in general” about TasP (91% HIV-positive vs. 69% HIV-negative men), men's articulation of their knowledge of TasP was poor – only 21% of HIV-positive and 13% of HIV-negative GBMSM demonstrated complete TasP knowledge in their short-answer definitions. The factor omitted most was “viral suppression,” perhaps suggesting a lack of understanding of the mechanism through which ART prevents illness among HIV-positive people as well as transmission. Although, with the open-ended nature of this question, participants may have assumed viral suppression was implied. Further, 13% of HIV-negative men and 5% of HIV-positive men described PrEP/PEP only, reflecting a general understanding of ART-based prevention approaches but highlighting a gap in knowledge on the essential differences between PrEP, PEP and TasP and underscoring a need to improve men's literacy of the various approaches [29]. Further, among those aware of TasP, only 64 and 41%, respectively, felt HIV treatment made the risk of transmitting or acquiring HIV “a lot lower,” despite a growing evidence base that suggests the high efficacy of this approach [30,31]. This echoes previous research showing that HIV-positive men and those engaging in practices that put them at an increased risk for infection are more likely to believe in the preventive benefits of ART [8]. Continued promotion of the individual health and preventative benefits of ART remains critical, particularly among HIV-negative and other communities of GBMSM who may be missed in current TasP promotional efforts.

The results of this study also shed light on how GBMSM access information related to TasP, with information sources varying considerably by HIV status. While doctors were the leading information source for HIV-positive GBMSM (44%), they were the least likely source for HIV-negative GBMSM (10%), highlighting how physicians can be important gatekeepers of information that they feel is relevant to their patients’ health. This is despite 95% of HIV-positive men and 68% of HIV-negative men being “out” to their family doctors. Notably, GBMSM were also unlikely to report learning about TasP from sex partners (10% HIV-positive men versus 17% HIV-negative men). These results may indicate challenges GBMSM have around participating in conversations with doctors and sex partners about HIV, sexuality, and ART-based prevention strategies such as TasP [32], particularly within a background of persistent stigma towards HIV-positive people and the risks of criminal charges related to HIV non-disclosure. This emphasizes the importance of continued work to de-stigmatize HIV, within which the negative impact of criminalization of HIV non-disclosure must be considered [33]. Community agencies (38% HIV-positive men vs. 25% HIV-negative men) and gay media (34% for both HIV-positive and HIV-negative men) were also key sources of information. Although not explored in this survey, HIV and sexually transmitted infections (STI) testing services as well as various online modes (e.g. mobile phone applications, and social media campaigns) may also be important population-based vehicles through which this kind of education could occur.

Overall, these findings indicate that generating and disseminating TasP messages cannot take a one-size-fits-all approach. Rather, it requires a consideration of the diversity of the target audience as well as gay men's health and media literacy (or the ways in which they use, interpret, and respond to information). This is consistent with previous research highlighting how effectively targeting HIV prevention messaging to diverse communities of GBMSM requires the development of a variety of health promotion messages at both an individual- and population-level, and that are also grounded in and culturally relevant to both venue/mode (e.g. Internet, bars, clinics) and person characteristics (e.g. age, culture, education levels) [29].

A study limitation is that we used baseline data collected from participants over a two-year period. Any potential shifts in TasP awareness and knowledge over this time will be investigated in future work. Further, participants’ definitions of TasP may not be a complete proxy for and likely under estimates their entire understanding of the concept, as the open-ended nature of the survey question may have precluded some individuals from demonstrating their full knowledge. More direct closed-ended questions specifically addressing each of the three identified components of TasP knowledge may actually have produced a more accurate assessment of men's knowledge of this concept. The study was strengthened by its use of RDS to develop weighted population estimates.

## Conclusions

To our knowledge, this is the first study to specifically report on TasP awareness and knowledge among GBMSM using a more representative sampling approach (i.e. RDS). For GBMSM to make use of TasP as a tool for their own health and the health of their communities, they must understand it. Health communication strategies relevant to diverse communities of GBMSM are critical to advancing TasP health literacy.
